# Enhanced segregation of concurrent sounds with similar spectral uncertainties in individuals with autism spectrum disorder

**DOI:** 10.1038/srep10524

**Published:** 2015-05-22

**Authors:** I-Fan Lin, Takashi Yamada, Yoko Komine, Nobumasa Kato, Makio Kashino

**Affiliations:** 1 NTT Communication Science Laboratories, NTT Corporation, Atsugi, Kanagawa, Japan; 2 Medical Institute of Developmental Disabilities Research, Showa University, Tokyo, Japan; 3 ATR Brain Information Communication Research Laboratory Group, Kyoto, Japan; 4 Department of Information Processing, Interdisciplinary Graduate School of Science and Engineering, Tokyo Institute of Technology, Yokohama, Kanagawa, Japan; 5 CREST, JST, Atsugi, Kanagawa, Japan

## Abstract

When acoustic signals from different sound sources are mixed upon arrival at the ears, the auditory system organizes these acoustic elements by their features. This study shows that individuals with autism spectrum disorder (ASD) performed better in terms of hearing a target sequence among distractors that had similar spectral uncertainties. Their superior performance in this task indicates an enhanced discrimination between auditory streams with the same spectral uncertainties but different spectro-temporal details. The enhanced discrimination of acoustic components may be related to the absence of the automatic grouping of acoustic components with the same features, which results in difficulties in speech perception in a noisy environment. On the other hand, the ASD group and the control group had similar performance in hearing a target sequence among distractors that had different spatial cues defined by interaural intensity differences.

Sounds help us know our environment, and the ability to differentiate sounds generated by different sources is not trivial. In a social environment, we usually encounter difficulties when trying to separate target speech from competitive speech. Autism spectrum disorder (ASD) is a neurodevelopmental disorder, and it has been suggested that the impaired social interaction and communication of individuals with ASD can be partly explained by the difficulties they experience in understanding target speech in a noisy environment^1^,^2^. Previous studies have shown that their difficulties in segregating the auditory target from distractors may be due to their deficit in hearing the target in the temporal dips of the distractors^1^,^2^ and in segregating auditory streams based on frequency separation^3^ and different spatial cues^4^,^5^,^6^. Here we investigated whether individuals with ASD had difficulty in integrating acoustic elements with similar features or in segregating acoustic elements with different features, and the features examined in this study were spectral uncertainties (i.e., jittered frequencies vs. non-jittered frequencies) and spatial cues (specifically, interaural intensity differences). In the auditory system, unwanted or ‘masking’ sounds compete with desired or ‘target’ sounds at various levels. The terms ‘energetic masking’ and ‘informational masking’ have been coined to describe the disruption of the auditory presentation of target sounds caused by masking sounds^7^. Whereas ‘energetic masking’ refers to the disruption in the auditory periphery, ‘informational masking’ refers to the disruption in any of several stages of processing beyond the auditory periphery. This study focused on auditory processes above the peripheral level to avoid energetic masking but below language-related processes to avoid the different orientation behaviors toward speech found in ASD^8^,^9^. The target sounds and interfering sounds (maskers) used in the experiment were composed of spectrally separated pure tones.

In the experiment, the threshold for detecting the target among the maskers was measured by using an adaptive staircase procedure with a 3-down 1-up rule. The target sequence always had jittered frequencies and was sent only to the right ear. The masking sequences either had jittered frequencies (i.e., the jittered conditions shown in Fig. 1A) or had the same frequencies (i.e., the non-jittered conditions shown in Fig. 1B). For the monotic conditions, the maskers were sent to the right ear, and the target and maskers shared the same spatial cues. For the diotic conditions, the maskers were sent to both ears, and the target and maskers had different spatial cues. Listeners should perform better under the non-jittered conditions than under the jittered conditions if they can segregate acoustic signals carrying different spectral uncertainties, and they should perform better under the diotic conditions than under the monotic conditions if they can segregate acoustic signals carrying different spatial cues. On the other hand, if the participants with ASD did not group acoustic signals with similar spectral uncertainties automatically, their performance for the jittered condition should be better than that of the control group.

## Results

**Figure 2:**
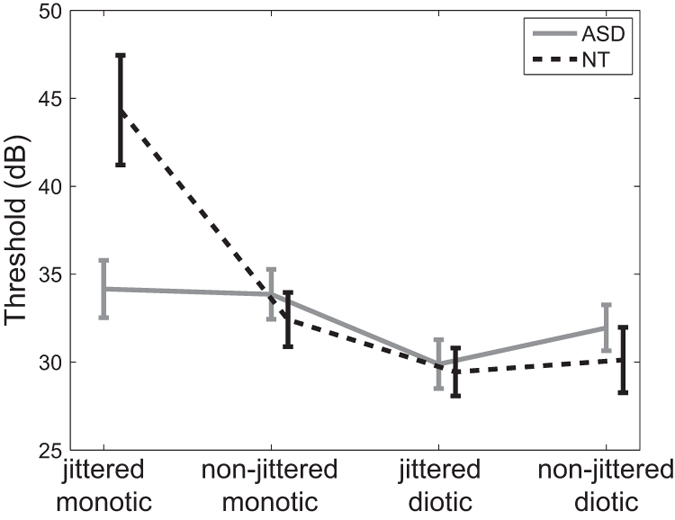
Target detection thresholds in the ASD and control groups for the four conditions, indicated as mean ± standard error. The maskers were sent to the right ear for the monotonic conditions or to both ears for the diotic conditions.

The cross-subject thresholds in the ASD and control groups for the four conditions are shown in Fig. 2. To investigate whether target-masker similarities in spectral uncertainty and the difference between spatial cues influenced the ASD subjects and neurotypical (NT) subjects in the same way, a three-way mixed-design ANOVA, with the between-subject factor as *Group* (ASD and NT) and the within-subject factor as *Spectral Uncertainty* (jittered and non-jittered frequencies) and *Spatial Cues* (monotic and diotic), was performed for the averaged thresholds in each condition. The ANOVA analysis was conducted with SPSS v.19 (IBM, USA).

In the ANOVA analysis, a significant effect of *Spectral Uncertainty* indicates masking release from target-masker dissimilarity with lower thresholds for the non-jittered condition compared to the jittered condition. On the other hand, a significant effect of *Spatial Cues* indicates masking release from the difference between spatial cues with lower thresholds for the diotic condition compared to the monotic condition. The ANOVA revealed a significant effect of *Spectral Uncertainty* (F(1,24) = 5.787, p = 0.024) and *Spatial Cues* (F(1,24) = 40.508, p < 0.001) but not *Group* (F(1,24) = 0.658, p = 0.425), and significant interactions between *Spectral Uncertainty* and *Group* (F(1,24) = 10.898, p = 0.003), *Spatial Cues* and *Group* (F(1,24) = 8.994, p = 0.006), and *Spectral Uncertainty* and *Spatial Cues* (F(1,24) = 21.549, p < 0.001) and among *Spectral Uncertainty* and *Spatial Cues* and *Group* (F(1,24) = 10.073, p = 0.004). The follow-up ANOVAs showed that the factor *Spectral Uncertainty* and the interaction between *Spectral Uncertainty* and *Group* was significant for the monotic conditions but not for the diotic conditions (while *Group* was not a significant factor for either monotic or diotic conditions), and the factors *Spatial Cues* and *Group* and the interaction between them were all significant for the jittered conditions but the factor *Spatial Cues* was the only significant one for the non-jittered conditions.

Driven by the hypothesis that the significant interaction between *Spectral Uncertainty* and *Group* for the monotic conditions and the significant interaction between *Spatial Cues* and *Group* for the jittered conditions were caused by the significantly different thresholds between groups for the jittered monotic condition, 4 between-group t-tests were conducted for the four conditions. These results confirmed that the significant difference between the performance observed in the ASD group and NT group mainly attributed to their significantly different thresholds for the jittered monotic condition (p = 0.018) but not other conditions (p > 0.05).

## Discussion

This study showed that both the ASD group and the control group were capable of segregating the target and the masker that carried different spatial information. On the other hand, for the monotic conditions, the significant difference between the jittered and non-jittered thresholds observed in the control group is consistent with the theory that informational masking is eliminated when the target and maskers have different spectral uncertainties^10^, but this difference was not observed in the ASD group. The fact that similar thresholds for the non-jittered monotic condition were observed in these two groups indicates that individuals with ASD had no difficulty in segregating the target and the masker that carried different spectral uncertainties. Nevertheless, the performance under the jittered monotic condition was significantly better in the ASD group than in the control group. This observation indicates that for individuals with ASD, the targets and maskers with similar spectral uncertainties were not mandatorily grouped together.

Previous studies show that individuals with ASD perform normally as regards sound localization in the horizontal plane but not in the vertical plane with background noise^11^. On the other hand, their spatial attention is affected when the target and distractors are presented from different directions at the same time^6^. These two previous studies did not differentiate the binaural cues used in horizontal sound localization. Another two studies that investigated sound segregation based on interaural time difference reported that individuals with ASD have reduced auditory evoked potentials related to pre-attentive target-masker segregation^4^,^5^. The combination of these studies with our findings suggests that individuals with ASD are likely to have a deficit in processing interaural time differences but not in processing interaural intensity differences. Nevertheless, the difference could also be explained by the single burst presented in the previous studies and the multiple bursts presented in this study because auditory object continuity (in the multiple-burst case) is known to increase selective auditory attention^12^.

On the other hand, the superior performance observed for the jittered monotic condition in the ASD group indicates that they were analytic listeners^13^, and this observation cannot be attributed to developmental delay because children are more susceptible to informational masking than adults^14^. Their superior performance in auditory stream segregation observed in this study might be attributed to (1) their enhanced discrimination between the target and masker, (2) their enhanced top-down target excitation, and (3) their enhanced distractor inhibition. The previous visual search study found that the superior performance of the ASD group is related to their superior discrimination between the target and distractors^15^. In this study, the target and maskers had the same spectral uncertainty under the jittered monotic condition and were grouped together in the control group, so their performance was disrupted by informational masking in this condition. However, if individuals with ASD could segregate the target and masker by the difference between their exact spectro-temporal structures, they could focus on the fixed spectral region for the target and had superior performance (as their performance in the non-jittered monotic condition). On the other hand, several studies indicate diminished top-down modulation in individuals with ASD^16^,^17^,^18^, and individuals with ASD have difficulty in filtering out the distractors^3^,^19^,^20^.

An increased prevalence of absolute pitch^21^,^22^ has been observed in individuals with ASD. In contrast, individuals with ASD have difficulty with processing complicated auditory stimuli^23^. There are two models that explain the seemingly contradictory auditory perception in autism: the weak central coherence theory and the enhanced perceptual functioning theory^24^,^25^. These two theories together describe the enhanced perception of local features and the diminished perception of global features. Nevertheless, the way to define ‘global’ features in the auditory domain is under debate. For example, the pitch contour is defined as a ‘global’ features in one study^26^ but a ‘local’ feature in another^27^, and in yet another study there are ‘global’ pitch contour and ‘local’ pitch contour^28^. The results of this study show that individuals with ASD were less affected by ‘global’ processes such as grouping auditory elements with similar spectral uncertainties. Since global processes are important for speech perception, the absence of these automatic processes in those with ASD might explain the observation that their superior performance in local processes seems to hinder their performance in speech perception^29^.

There have been few studies exploring why individuals with ASD have difficulty in isolating target speech from interfering sounds in a social environment. Our observation suggests that the absence of automatic grouping processes, which may be related to enhanced discrimination of acoustic components with similar features, may explain their difficulty in segregating target speech and background sounds. Speech requires the integration of acoustic elements across frequencies and time, especially when it is disrupted by background noise. If those with ASD have encountered difficulties in hearing target speech in a social environment since childhood because of the absence of automatic grouping processes, they may also encounter difficulties in communication and social interaction. In a clinical setting, these findings provide a possible early detection and intervention approach with respect to the difficulties in social interaction and communication exhibited by those with ASD, especially for those suffering from hearing difficulties without a detectable deficit in their peripheral auditory system.

## Methods

### Participants

Thirteen high-functioning adults with autism (3 females) and 13 NT subjects (5 females) were matched by age (mean±SD ASD group: 28 ± 7.47, NT group: 26.85 ± 5.23) and IQ (mean±SD ASD group: 108.62 ± 10.45, NT group: 114.31 ± 11.06) for both VIQ (mean±SD ASD group: 112.69 ± 10.7, NT group: 117.62 ± 11.74) and PIQ (mean±SD ASD group: 101.85 ± 12.16, NT group: 106.92 ± 11.89). All participants were evaluated regarding their autistic traits by autism spectrum quotient (mean±SD ASD group: 37.15 ± 4.32, NT group: 18.54 ± 5.19). All the participants performed normally in an audiometric test (hearing levels <20 dB from 0.25 to 8 kHz). All of the participants were naive as to the purposes of the study. All procedures were conducted in accordance with the Declaration of Helsinki and approved by the ethics committee of the NTT Communication Science Laboratories. The participants were paid for their time and gave their informed consent prior to their participation.

ASD participants were recruited from outpatient units of the Karasuyama Hospital, Tokyo, Japan. The diagnosis of ASD was based on a consensus reached by three experienced psychiatrists and one psychologist according to the criteria of Diagnostic and Statistical Manual of Mental Disorders (DSM-IV-TR), fourth edition^30^, after two detailed interviews conducted independently by a psychiatrist and a clinical psychologist belonging to the team at the hospital that included the participant’s developmental history, present illness, past history, and family history. They also confirmed that none of the participants with ASD met the DSM-IV criteria for any other psychiatric disorders (e.g., mood disorders, schizophrenia, anxiety disorders, or substance-related disorders). In addition, the diagnosis was reconfirmed at least after two months later.

### Auditory stimuli

The experiment was conducted in a sound-insulated booth. Auditory stimuli were generated by MATLAB (7.10.0) at a 20-kHz sampling rate and low-pass filtered at 7.5 kHz. The auditory stimuli were then processed by an audio interface (M-AUDIO FAST-TRACK PRO) and then sent to headphones (Senheiser HDA200).

The target sequence consisted of eight 60-ms pure tones (with 10-ms ramps) that were chosen randomly on a logarithmic frequency scale from 848 to 1180 Hz. Each of the masker sequences also consisted of eight 60-ms pure tones (with 10-ms ramps) that were chosen randomly on a logarithmic frequency scale from 200 to 5000 Hz with the exclusion of the protected spectral region from 800 to 1250 Hz. In total the duration of the auditory stimuli in each interval was 480 ms. Each component in the masker was 57 dB SPL, and the total sound level was around 66 dB SPL.

The frequencies of the target and masker components were randomly selected for each interval. By selecting the frequencies of the masker components outside a protected spectral region, although energetic masking was not completely avoided, it was minimized. For ‘holistic’ listeners, Durlach *et al.* shows that subjects are severely distracted by the masker and find it difficult to discern the target even though there is little masker energy in the frequency region of the target^10^.

### Procedure

Each trial consisted of two intervals, separated by a 1-s silent gap. In one of the two intervals, chosen at random to be the first or the second with equal probability, the target was presented with the masker; in the other interval, only the masker was presented. Listeners were instructed to select the interval in which the target was presented. Once listeners had given their answers, feedback was provided in the form of a message displayed on the computer screen (‘correct’ or ‘incorrect’).

The threshold of target detection was measured by using an adaptive staircase procedure with a 3-down 1-up rule, which tracks the point corresponding to 79.4% correct on the psychometric function^31^. A total of four adaptive tracks, with a minimum of 50 trials and 12 reversals each, were obtained for every condition. In the beginning of each adaptive track, the sound level of the components in the target was set to be 57 dB SPL. The sound level of the target was decreased or increased by 8 dB before the second reversal, and then the sound level was decreased or increased by 4 dB before the fourth reversal. After the fourth reversal, the sound level of the target was decreased or increased by 2 dB. The sound level of the target in the last six reversals was averaged as the threshold in that adaptive track. While the first adaptive track was served as practice, the average of the thresholds measured in the last three adaptive tracks in each condition was used for further analysis.

## Additional Information

**How to cite this article**: Lin, I. F. *et al.* Enhanced segregation of concurrent sounds with similar spectral uncertainties in individuals with autism spectrum disorder. *Sci. Rep.* **5**, 10524; doi: 10.1038/srep10524 (2015).

## Figures and Tables

**Figure 1:**
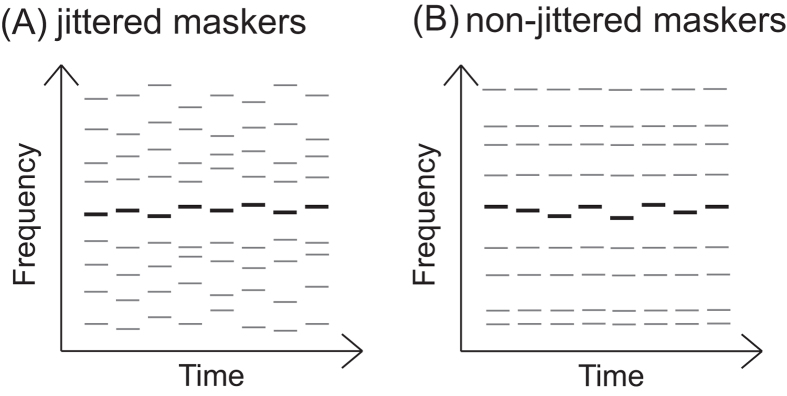
The auditory stimuli contained one target sequence (black lines) and eight masker sequences (gray lines). The target sequence always had jittered frequencies within a fixed protected region. (**A**) The masker sequences had jittered frequencies outside the protected region for the jittered conditions. (**B**) The masker sequencies had fixed frequencies outside the protected region for the non-jittered conditions.
